# Supplementary material for: Cranial Base Synchondrosis Lacks PTHrP-Expressing Column-Forming Chondrocytes
Source: Int J Mol Sci. 2022 Jul 17;23(14):7873. doi: 10.3390/ijms23147873 (PMC9315528; doi:10.3390/ijms23147873)
Supplement: Supplementary file 1 [file ijms-23-07873-s001.zip › ijms-1799395-supplementary.pdf]

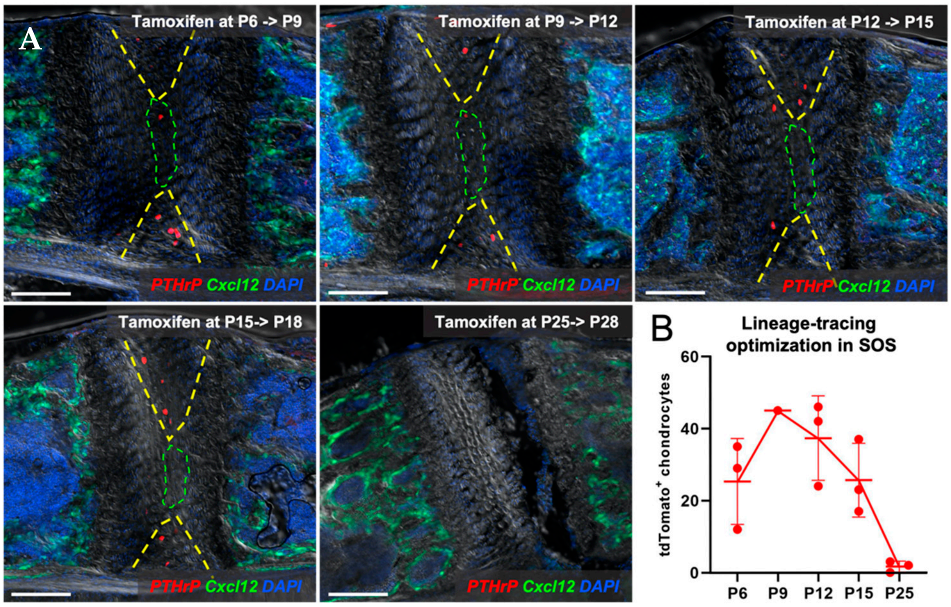

Figure S1. *Pthrp-creER* marks chondrocytes in the lateral wedge-shaped area of the synchondrosis. (**A**) *Pthrp-creER*; *R26RtdTomato* mice were pulsed at P6, P9, P12, P15 and P25, and the SOS was analyzed at 72 h later at P9, P12, P15, P18 and P28, respectively. Yellow dotted lines: wedge-shaped areas, green dotted lines: central hypertrophic zone. Arrowheads: tdTomato$^+$ cells in the resting zone. Red: tdTomato, blue: DAPI, gray: DIC, green: *Cxcl12-GFP*. Scale bars: 100 μm. (**B**) Quantification of tdTomato$^+$ chondrocytes in SOS. x-axis: day of tamoxifen pulse. y-axis: tdTomato$^+$ chondrocytes. The number of tdTomato$^+$ chondrocytes was counted at 72 hours after the initial pulse. *n* = 1–3 for each group/timepoint. Data are present as the mean ± S.D.
